# *Anopheles* diversity, biting behaviour and transmission potential in forest and farm environments of Gia Lai province, Vietnam

**DOI:** 10.1186/s12936-023-04631-1

**Published:** 2023-07-05

**Authors:** Nguyen Van Dung, Nguyen Quang Thieu, Hoang Dinh Canh, Bui Le Duy, Vu Viet Hung, Nguyen Thi Hong Ngoc, Nguyen Vu Tuyet Mai, Ngo Thi Van Anh, Le Duy Son, Win Han Oo, Win Htike, May Chan Oo, Naw Hkawng Galau, Kaung Myat Thu, Aung Khine Zaw, Ei Phyu Htwe, Julia C. Cutts, Ellen A. Kearney, Bangyuan Wang, Paul A. Agius, Freya J. I. Fowkes, Katherine O’Flaherty

**Affiliations:** 1 National Institute of Malariology, Parasitology and Entomology, Hanoi, Vietnam; 2 Health Poverty Action Vietnam, Hanoi, Vietnam; 3 Health Security Program, Burnet Institute Myanmar, Yangon, Myanmar; 4 Disease Elimination Program, Burnet Institute, Melbourne, Australia; 5 Department of Medicine, The University of Melbourne, Melbourne, Australia; 6 Melbourne School of Population and Global Health, The University of Melbourne, Melbourne, Australia; 7 Health Poverty Action, London, UK; 8 Department of Epidemiology and Preventive Medicine, Monash University, Melbourne, Australia; 9 Biostatistics Unit, Faculty of Health, Deakin University, Melbourne, Australia; 10 Department of Infectious Diseases, Monash University, Melbourne, Australia

**Keywords:** Anopheles, Diversity, Plasmodium, Malaria, Vector, Vietnam

## Abstract

**Background:**

Despite recent reductions in Vietnam, malaria transmission persists in some areas in forests and farmlands where a high density of *Anopheles* mosquitoes relative to other environments occurs. To inform effective malaria control measures, it is important to understand vector bionomics and the malaria transmission role of *Anopheles* spp. in the highland regions of Vietnam. This study was conducted to quantify the abundance, composition and biting behaviour of the *Anopheles* mosquito population, and the proportion of *Plasmodium* spp. infected mosquitoes collected from forest and agricultural farm sites in Gia Lai province, Vietnam.

**Methods:**

Forest and agricultural farm sites in Gia Lai province were selected for mosquito collections (total eight sites). Mosquito collection was performed by Human-baited Double Net Trap (HDNT), animal-baited traps (ABT) using cattle, and CDC light traps. Captured mosquitoes were identified morphologically, and salivary glands of *Anopheles* mosquitoes were examined for sporozoites using microscopy. *Plasmodium* infection was determined by Polymerase Chain Reaction (PCR), and identification of blood meal type was determined by PCR and diffuse serum agglutination assay.

**Results:**

A total of 1815 *Anopheles* mosquitoes belonging to 19 species were collected by ABT (n = 1169), HDNT (n = 471) and CDC light trap (n = 175). *Anopheles* abundance and diversity varied by district and environment. Capture by HDNT of *Anopheles* of vectorial concern was observed between early evening and early morning. *Plasmodium vivax* infection was determined by PCR in two *Anopheles dirus* specimens captured by HDNT in forest sites. Blood from a range of hosts could, including human blood, could be detected in species considered primary and secondary vectors *An. dirus*, and *Anopheles aconitus*, and *Anopheles maculatus*, respectively.

**Conclusions:**

A low number of *Anopheles* spp. considered primary vectors of concern and very low numbers of *Plasmodium* spp. infected *Anopheles* mosquitoes were captured at the end of the rainy season in the Central Highlands of Vietnam. However, capture species of vectorial concern by HDNT throughout the early to late evening demonstrates that use of additional personal protective measures could supplement current preventative measures, such as bed nets to prevent exposure to vectors of concern in this region.

**Supplementary Information:**

The online version contains supplementary material available at 10.1186/s12936-023-04631-1.

## Background

Between 2000 and 2021, malaria case incidence and mortality significantly declined in Vietnam, from 74,316 to 459 confirmed cases (99.4% reduction) [1]. The declining trend in incidence was consistent across all provinces in Vietnam and largely attributed to the distribution and uptake of insecticide-treated nets (ITNs), access to early diagnosis and treatment and high coverage of artemisinin-based combination therapy [1, 2]. With zero reported deaths due to malaria since 2019 and the majority of provinces now certified malaria-free [3], Vietnam is progressing towards its strategic goal of malaria elimination by 2030 [4].

In Vietnam, human malaria transmission is mostly concentrated in mountainous, forested areas in southern and central provinces [1]. Despite recent reductions in malaria cases and fatalities, malaria persists in some areas of the Central Highland and Southeast areas of Vietnam [5, 6]. Many of these cases occur in local residents sleeping in forested or farmed areas [6, 7], where use of ITNs is lower compared with local villages [5], and the density of *Anopheles* mosquitoes is usually high [8].

There are two primary and at least six secondary *Anopheles* species which transmit malaria within Vietnam. The primary malaria vector *Anopheles dirus* is only found in the mountainous areas from the 20º North latitude (South Thanh Hoa) to the South of Vietnam, while the other primary vector *Anopheles minimus* is distributed mainly in mountainous, highland and midland areas, as is secondary vector *Anopheles maculatus*. Recommended vector control interventions within Vietnam include indoor residual spraying, and use of ITNs which are distributed free of charge [4]. However, *Anopheles* vectors within the region display varied biting and resting behaviours that may circumvent these protective measures such as early and outdoor biting [8, 9]. The diversity and host-seeking behaviour of *Anopheles* spp. mosquitoes in Gia Lai Province has recently been investigated [8], however, regular monitoring of *Anopheles* spp. transmission potential and biting preferences is also required and is essential to inform effective vector prevention measures where populations working or residing outdoors are at greater risk of vector exposure. Therefore, mosquito collections were undertaken in two districts of Gia Lai province, Vietnam, to quantify the abundance, composition and distribution of the mosquito population, mosquito biting behaviour and the proportion vectors infected with *Plasmodium* spp. parasites in farm and forest sites.

## Methods

### Study setting

**Fig. 1:**
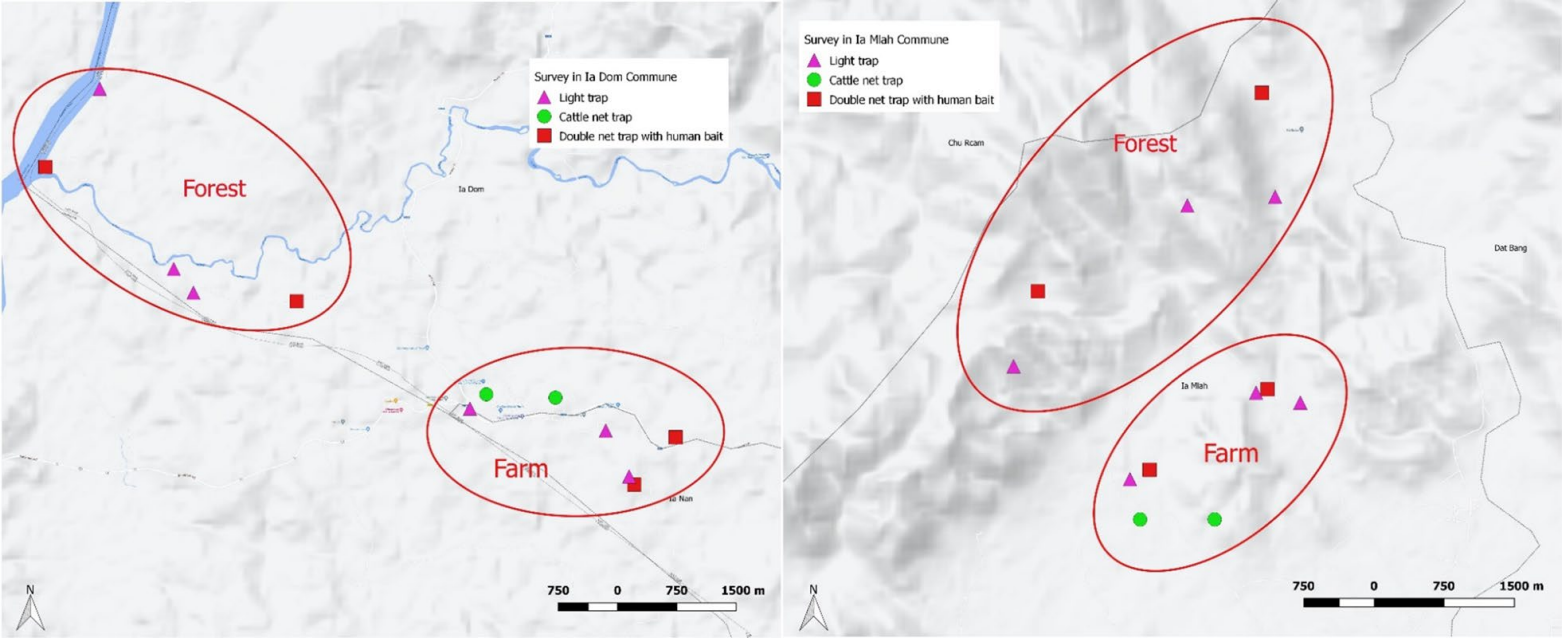
Location of different capture methods in farm and forest sites in Ia Dom commune, Duc Co District and Ia Mlah commune, Krong Pa District in Gia Lai Province, Vietnam. Animal baited traps and human baited double net traps were located at least 600 m apart (range: 0.6–2.5 km), and light traps were located at least 100 m away from other collection types

Mosquito collections were made in eight sites representing forested and farm environments of Duc Co and Krong Pa districts in Gia Lai province, Vietnam. Gia Lai province is in the Central Highlands region of Vietnam (Additional file 1: Fig. S1). The total area is 724.28 km², and to the west is the 35 km long Vietnam - Cambodia border line bordering with Ratanakiri province, Cambodia. Gia Lai is a remote highland district with mountainous terrain and difficult travel conditions. There are two seasons; the rainy season starts from May to October and the dry season is from November to April. September is the month with the highest rainfall. The climatic characteristics of Krong Pa district are somewhat different from other regions in Gia Lai and the Central Highlands (including Duc Co district), as it is tropical to slightly dry due to the mountainous topography, which shields the wind direction from east and southwest. One commune from each district was selected for mosquito collection surveys. Ia Dom commune in Duc Co district and Ia Mlah commune in Krong Pa district are malaria endemic areas where residents frequently sleep in the forest and the farm. Within each commune, two farm and two forested locations were selected for collections (total eight sites, Fig. 1). Ia Dom commune has a low hilly terrain. There are two seasons; rainy from May to November and dry from December to April. The climate is considered tropical monsoon like and the average rainfall is 1500–1600 mm. The commune includes seven villages with an approximate total population of 8400 people. The majority of the population is aged between 18 and 60 years (50%), 30% are aged under 18 years and 20% are aged over 60 years. Approximately 45% of working occupants regularly enter farms and forests for work. Farm locations where traps were located included cashew plantations with surrounding small flowing streams, and forests consisted of mostly bamboo and shrubs with small flowing streams. In Ia Dom, both *Plasmodium falciparum* and *Plasmodium vivax* cases have been reported, with all cases preceding this survey being *P. falciparum* (three *P. falciparum* cases reported in 2020). Ia Mlah commune has mountainous and low lying hilly terrain. There are two seasons; rainy from May to October and dry from November to April. The climate is considered tropical and slightly dry, and the average rainfall is 1200 mm. The commune includes four villages with an approximate total population of 4200 people. The majority of the population is aged between 18 and 60 years (56%), 38% are aged under 18 years and 6% are aged over 60 years. Approximately 85% of working occupants regularly enter farms and forests for work. Farm locations where traps were located included cashew and cassava plantations with surrounding small flowing streams, and forests consisted of mostly bamboo and shrubs with small flowing streams. In Ia Mlah, reported cases of malaria were somewhat higher than Ia Dom preceding this survey. Both *P. falciparum* and *P. vivax* cases have been reported, the majority being *P. falciparum* (25 and 15 *P. falciparum* cases and 13 and 2 *P. vivax* cases reported in 2020 and 2021, respectively).

### Mosquito collection

At each site, mosquito collection by Human baited Double Net Trap (HDNT) was performed (total eight traps) by teams of three volunteer collectors (total 24 collectors), each performing one eight-hour shift (06:00–14:00, 14:00–22:00 or 22:00–06:00) per day for twelve consecutive days. Residents aged between 18 and 60 years old, in general good health were eligible to volunteer as mosquito collectors. Mosquito collectors completed a one-day training on the procedure, data collection and sample storage methods prior to collection. In all sites, an experienced mosquito collector oversaw all collections made by volunteers. Mosquitoes were captured by HDNT as described by Tangena et al. [10] with the following modifications. Traps were set up outdoors, with the outer net raised 30 cm from the ground to allow mosquito entry. Inner and outer nets were separated by a 50 cm gap, and the collector entered the gap every 15 min to perform collections between the two nets, as well as on the outer side of the larger net. Collectors wore protective clothing (pants, long sleeved shirts and boots) and rotated shifts every eight hours for a total 24 h. Collections from outside the outer net were also made using a torch light and collection tube. All specimens were stored separately according to hour of collection. Animal baited traps (ABT) using cattle were set up in farm sites in each commune and placed between 0.6 and 2.5 km from the closest HDNT (two traps per district, total four ABT). Briefly, ABT were hung outside in farm sites leaving an approximate 30 cm gap between the net and the ground. Cattle baits were tied in the centre of the net. Mosquito collections were performed hourly by a single volunteer collector from 18:00 until 06:00 h for 12 days. Using a flashlight, collectors captured mosquitoes on the animal and trapped around the inner side of the net into collection tubes. Collectors were provided with protective clothing. CDC light traps (light lure only) were suspended 1.5 m above ground in three forest and three farm sites per commune (six traps per district, total 12 CDC light traps) at least 100 m apart from other mosquito collection activities (i.e., HDNT). Light attracted mosquitoes were trapped by fan-forced suction. Traps were set from 18:00 until 06:00 h for 12 days, and trapped specimens were collected and stored according to collection site at the end of each collection.

### Mosquito identification and diversity indices

**Fig. 2:**
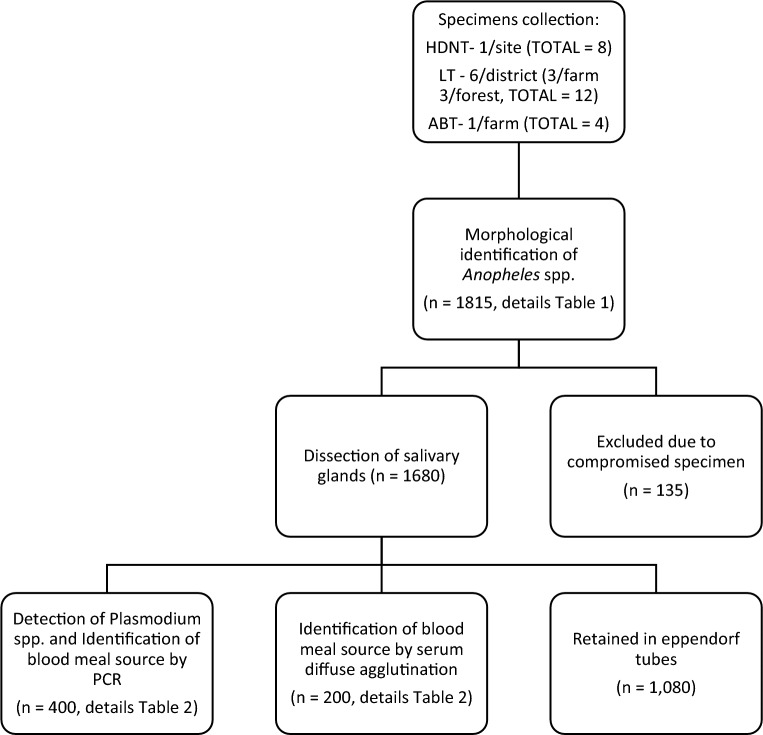
Summary of specimen collection and sample flow. HDNT; Human-baited double net trap, LT; Light trap, ABT; Animal baited trap

All captured mosquitoes were identified using a stereomicroscope and morphological identification keys (Keys to the anopheline mosquitoes of Vietnam—National Institute of Malariology Parasitology and Entomology Vietnam). Species diversity was determined by calculating the Shannon’s diversity and equitability indexes [11]. Subsequent analyses of captured specimens are described below and summarized in Fig. 2.

### **Identification of*Plasmodium*spp. infection**

*Anopheles* mosquitoes were dissected and the salivary glands were examined for sporozoites by microscopy in the commune health station (n = 1680). Dissected head/thoraces and midguts were preserved in beem capsules with silica gel for transport and subsequent analyses. Identification of *Plasmodium* spp. was performed in a subset of specimens (n = 400), with priority of inclusion given to specimens collected by HDNT. DNA was extracted from dissected head/thoraces using the Biofact genomic DNA Prep kit (BioFact South Korea) as per manufacturers instructions. qPCR assays to detect *Plasmodium* spp. were undertaken as summarised in the Supplementary Material (Additional file 1: Tables 1 and 2), using Applied Biosystems 7500 Real Time PCR System (and 2.06 Software) instrumentation. All results with Ct ≤ 40 were considered positive.

### Host blood origin identification

To understand the host biting preferences in captured specimens, determination of host blood origin was performed in separate subsets of specimens by PCR, and by serum diffuse agglutination for specimens with detectable blood in the midgut (Fig. 2). DNA was extracted from dissected midguts of *Anopheles* spp. mosquitoes (n = 400) and used to determine the origin of host blood (human, bovine, porcine) by PCR according to previously described methods [12]. Host blood origin (human, bovine, canine, poultry) was also determined in a subset of *Anopheles* spp. mosquitoes recorded as having visible blood in the midgut (n = 200) by serum agglutination assay as previously described [13].

## Results

### **Species composition, density and diversity of captured*Anopheles*mosquitoes**

Mosquito collections were made in eight sites representing forested and farm environments of Duc Co and Krong Pa districts in Gia Lai province, Vietnam (Fig. 1). Mosquito collections were carried out over 12 consecutive days in late November/early December 2021. Overall, a total of 2927 mosquitoes were collected from 12 collection nights belonging to the genera *Aedes* spp. (n = 49), *Anopheles* spp. (n = 1815), *Culex* spp. (n = 1060) and *Mansonia* spp. (n = 3) (Additional file 1: Table 3). From the 1815 *Anopheles* spp. mosquitoes collected, a total of 19 species were identified by morphology (Table 1). The majority of *Anopheles* spp. specimens were collected from ABT (n = 1169), followed by HDNT (n = 471) and CDC light trap (n = 175).

**Table 1:** Composition and density of Anopheles spp. mosquitoes by district, site and capture method

*Anopheles* spp.	Ia Dom commune, Duc Co district - N mosquitoes (density)					Ia Mlah commune, Krong Pa district - N mosquitoes (density)				
	Forest		Farm			Forest		Farm		
	HDNT	LT	HDNT	LT	ABT	HDNT	LT	HDNT	LT	ABT
Primary vector species										
* An. dirus*		1 (0.03)				20 (0.83)	8 (0.22)	3 (0.13)	8 (0.22)	
* An. minimus*	1 (0.04)		15 (0.63)	14 (0.39)	39 (1.63)			3 (0.13)		
Secondary vector species										
* An. aconitus*	19 (0.79)	15 (0.42)	28 (1.17)	27 (0.75)	154 (6.42)	111 (4.63)	35 (0.97)	188 (7.83)	25 (0.69)	192 (8.00)
* An. barbirostris*			2 (0.08)	3 (0.08)	11 (0.46)					11 (0.46)
* An. jeyporiensis*										2 (0.08)
* An. maculatus*	19 (0.79)	4 (0.11)	9 (0.38)	7 (0.19)	86 (3.58)	7 (0.29)	1 (0.03)	11 (0.46)	1 (0.03)	3 (0.13)
* An. peditaeniatus*	10 (0.42)	3 (0.08)	2 (0.08)	3 (0.08)	43 (1.79)					
* An. philippinnensis*			3 (0.13)	1 (0.03)	79 (3.29)				1 (0.03)	
* An. sinensis*	4 (0.17)	1 (0.03)	3 (0.13)	2 (0.06)	18 (0.75)					12 (0.50)
* An. vagus*					3 (0.13)	1 (0.04)		1 (0.04)	2 (0.06)	69 (2.88)
Other species										
* An. anularis*					1 (0.04)					
* An. argyropus*					6 (0.25)					
* An. crawfordi*					4 (0.17)					
* An. jamesi*				1 (0.03)	63 (2.63)					32 (1.33)
* An. kawari*					84 (3.50)					
* An. kochi*					18 (0.75)				1 (0.03)	19 (0.79)
* An. nigerimus*					1 (0.04)					
* An. splendidus*		1 (0.03)	2 (0.08)	7 (0.19)	125 (5.21)	2 (0.08)		7 (0.29)	3 (0.08)	75 (3.13)
* An. tessellatus*					3 (0.13)					16 (0.67)
No. of species	5	6	8	9	17	5	3	6	7	10

Density = number of mosquitoes/number of collection sources/number of days, number of collection sources per commune: HDNT = 4, ABT = 2, LT = 6, days: HDNT = 24 h, ABT = 12 h, LT = 12 h

HDNT: human baited double net trap, ABT: animal baited trap, LT: light trap

Overall, a greater number of specimens and species diversity was observed in Ia Dom (n = 945, n species = 18, Shannon’s diversity index (H) = 2.24 and equitability index (E_H)_ = 0.77). compared with Ia Mlah commune (n = 870, n species = 13, H = 1.40 E_H_=0.55), in farm sites (n = 1552, n species = 20, H = 2.12 E_H_=0.71) compared with forests (n = 263, n species = 8, H = 1.07 E_H_=0.52), and capture by ABT (n = 1169, n species = 19, H = 2.27 E_H_=0.77) compared with HDNT (n = 471, n species = 10, H = 1.05 E_H_=0.46) and CDC light trap (n = 175, n species = 12, H = 1.53 E_H_=0.61) (Table 2). In both communes, the greatest number and density of collected specimens belonged to *Anopheles aconitus* species, irrespective of site or trapping method. *An. dirus* and *An. minimus* species, commonly regarded as primary vector species in the surveyed region, were found in comparatively low numbers in in both districts comparative to other species (Table 1). Species composition was consistent between HDNT and CDC light traps conducted in farm and forest sites within each commune, however the number and diversity of species captured by each method was greater in Ia Dom compared with Ia Mlah commune (Tables 1 and 2).

**Table 2:** Diversity of captured Anopheles spp. by commune, environment and trapping method

Overall									
Ia Dom, Duc Co	n = 945, n species = 18					H = 2.24 E_H_=0.77			
Ia Mlah, Krong Pa	n = 870, n species = 13					H = 1.40 E_H_=0.55			
Farm	n = 1,552, n species = 20					H = 2.12 E_H_=0.71			
Forest	n = 263, n species = 8					H = 1.07 E_H_=0.52			
HDNT	n = 471, n species = 10					H = 1.05 E_H_=0.46			
LT	n = 175, n species = 12					H = 1.53 E_H_=0.61			
ABT	n = 1,169, n species = 19					H = 2.27 E_H_=0.77			
By Commune									
	Ia Dom, Duc Co								
Farm	n = 867, n species = 17		H = 2.25 E_H_=0.79			n = 685, n species = 13		H = 1.47 E_H_=0.58	
Forest	n = 78, n species = 7		H = 1.36 E_H_=0.70			n = 185, n species = 5		H = 0.69 E_H_=0.43	
HDNT	n = 117, n species = 8		H = 1.62 E_H_=0.78			n = 354, n species = 6		H = 0.63 E_H_=0.35	
LT	n = 90, n species = 10		H = 0.68 E_H_=0.29			n = 85, n species = 7		H = 0.96 E_H_=0.49	
ABT	n = 738, n species = 17		H = 2.30 E_H_=0.80			n = 431, n species = 10		H = 1.66 E_H_=0.72	
	Ia Dom, Duc Co					Ia Mlah, Krong Pa			
	Forest		Farm			Forest		Farm	
HDNT	n = 53, n species = 5	H = 1.32 E_H_=0.82	n = 64, n species = 8	H = 1.59 E_H_=0.76		n = 141, n species = 5	H = 0.71 E_H_=0.44	n = 213, n species = 6	H = 0.52 E_H_=0.29
LT	n = 25, n species = 6	H = 1.24 E_H_=0.69	n = 65, n species = 9	H = 1.70 E_H_=0.77		n = 44, n species = 3	H = 0.58 E_H_=0.53	n = 41, n species = 7	H = 1.23 E_H_=0.63
ABT	-	-	n = 738, n species = 17	H = 2.27 E_H_=0.80		–	-	n = 431, n species = 10	H = 1.66 E_H_=0.72

ABT; Animal baited trap, HDNT; Human baited double net trap, LT; Light trap, H = Shannon diversity index, E_H_=Shannon equitability index

In Ia Dom commune, the number of specimens and species diversity was greater in farm (n = 867, n species = 17, H = 2.25 E_H_=0.79) compared to forest sites (n = 78, n species = 7, H = 1.36 E_H_=0.70), likely because ABT were not performed in forests and resulted in the greatest number of specimen collections and had the greatest diversity (n = 738, n species = 17, H = 2.30 E_H_=0.80) compared to HDNT (n = 117, n species = 8, H = 1.62 E_H_=0.78) and CDC light traps (n = 90, n species = 10, H = 0.68 E_H_=0.29) (Table 2). The number and diversity of *Anopheles* mosquitoes collected were similar between HDNT conducted in the farm (n = 64, n species = 8, H = 1.59 E_H_=0.76) and forest sites (n = 53, n species = 5, H = 1.32 E_H_=0.82). In farm sites, a total of eight *Anopheles* species were captured by HDNT, the highest number of collections were made for *An. aconitus* (n = 28) (Table 1). In forest sites, five *Anopheles* species were captured by HDNT, the highest number of collections were made for *An. aconitus* and *An. maculatus* (n = 19, both species). In both farm and forest sites, the number of collected *An. aconitus* was highest of all *Anopheles* captured by CDC light trap (Table 1). Only a single *An. dirus* specimen was captured in Ia Dom by CDC light trap in the forest.

In Ia Mlah commune, Krong Pa district, the number of specimens collected, and number of *Anopheles* species identified was smaller compared with Duc Co district (Table 1). However, similarly to Ia Dom, the number of specimens and species diversity was greater in farm (n = 685, n species = 13, H = 1.47 E_H_=0.58) compared to forest sites (n = 185, n species = 5, H = 0.69 E_H_=0.43), and ABT resulted in the greatest number of specimen collections and had the greatest diversity (n = 431, n species = 10, H = 1.66 E_H_=0.72) compared to HDNT (n = 354, n species = 6, H = 0.63 E_H_=0.35) and CDC light traps (n = 85, n species = 7, H = 0.96 E_H_=0.49) (Table 2). The diversity of *Anopheles* mosquitoes collected were similar between HDNT conducted in the farm (n = 213, n species = 6, H = 0.52 E_H_=0.29) and forest sites (n = 141, n species = 5, H = 0.71 E_H_=0.44), however, the overall number of Anopheles collected by HDNT was greater in farm sites. In the farm, a total of six *Anopheles* species were captured by HDNT, and the highest number of collections was made for *An. aconitus* (n = 188) (Table 1). In the forest, five *Anopheles* species were captured by HDNT, and the highest number of collections was for *An. aconitus* (n = 111). In both farm and forest sites, the number of collected *An. aconitus* was highest compared to other species (Table 1).

### **Host-seeking and biting behaviours of*Anopheles*mosquitoes**

**Fig. 3:**
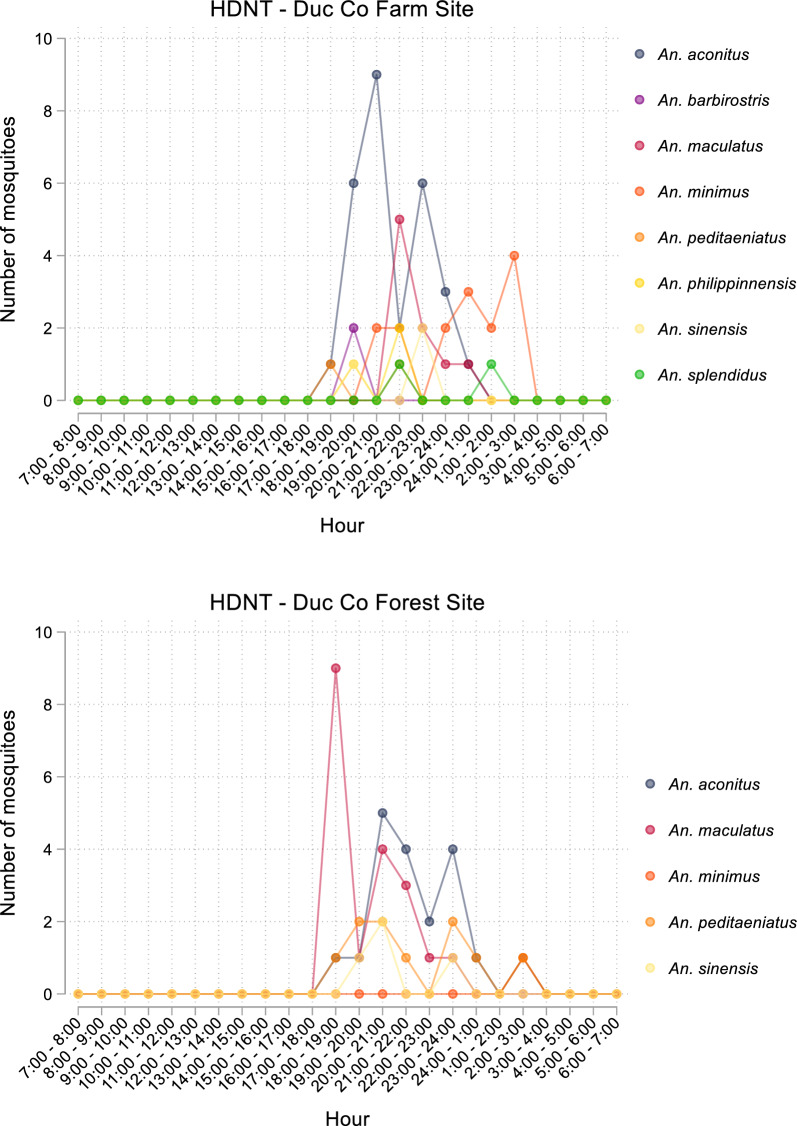
Number of Anopheles mosquitoes collected by HDNT in farm and forest sites in Duc Co District by species

**Fig. 4:**
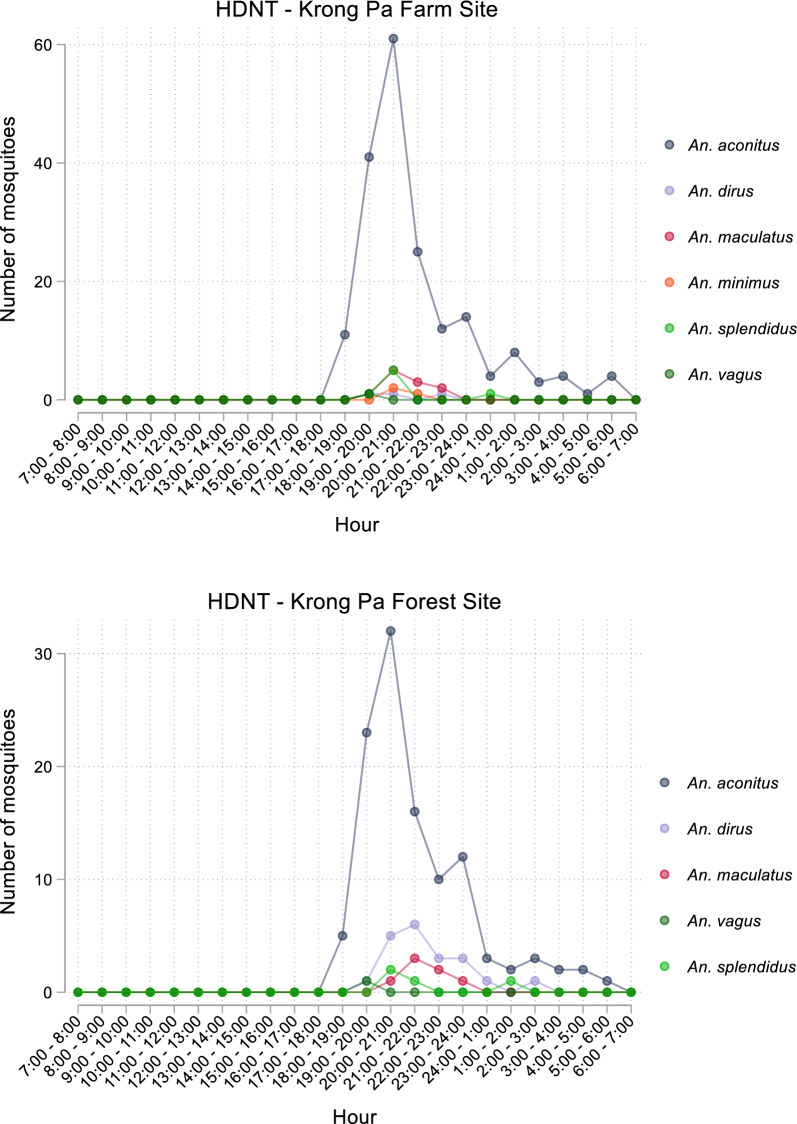
Number of Anopheles mosquitoes collected by HDNT in farm and forest sites in Krong Pa District by species

In both communes, capture times were consistent across collection nights (Additional file 1: Figs. S2 and S3), and species considered vectors of concern *An. minimus, An. dirus* and *An. maculatus* were captured in lower numbers comparative to *An. aconitus*. In Ia Dom commune, Duc Co district, capture of *Anopheles* spp. by HDNT occurred between 17:00 and 04:00 (Fig. 3). *Anopheles aconitus* was captured in farm sites between 18:00 and 01:00, and in forest sites between 18:00 and 03:00. In both farm and forest sites, the greatest number of *An. aconitus* collections was made between 20:00 and 21:00. In Ia Mlah commune, Krong Pa district, capture of *Anopheles* spp. by HDNT occurred between 18:00 and 06:00 (Fig. 4). *Anopheles aconitus* was captured in farm and forest sites between 18:00 and 06:00, with the greatest number of collections made between 20:00 and 21:00.

To understand the host biting preferences in captured specimens, determination of host blood origin was performed in separate subsets of specimens by PCR (n = 400), and by serum diffuse agglutination for specimens with detectable blood in the midgut (n = 200) (Table 3). By both methods, cattle blood was detected in the majority of species (14/15) and specimens (243/386) tested (Table 3). Human blood could be detected in specimens identified as *An. aconitus, An. dirus, Anopheles jamesii, An. maculatus, Anopheles sinensis, Anopheles splendidus* and *Anopheles vagus* (Table 3). In *An. dirus* specimens with a visible blood meal (n = 4), the origin of blood was confirmed as exclusively human by serum diffuse agglutination, however, multiple sources of blood meal were identified in separate *An. dirus* specimens assessed by PCR. No individual specimen was found to have a blood meal originating from more than one origin species.

**Table 3:** Host blood origin in Anopheles spp. determined by PCR and diffused serum agglutination

*Anopheles* spp.	Host blood origin (n positive)			
Determined by PCR (n = 400)				
	Human	Cattle	Pig	Negative
* An. aconitus* (n = 217)	0	41	38	138
* An. dirus* (n = 36)	2	8	25	1
* An. jeyporiensis* (n = 2)	0	1	1	0
* An. maculatus* (n = 56)	0	4	23	29
* An. minimus* (n = 79)	0	15	27	37
* An. sinensis* (n = 1)	0	0	1	0
* An. splendidus* (n = 9)	0	0	0	9
Determined by diffused serum agglutination (n = 200)				
	Human	Cattle	Canine	Poultry
* An. aconitus* (n = 100)	23	72	9	1
* An. barbirostris* (n = 1)	0	1	0	0
* An. dirus* (n = 4)	4	0	0	0
* An. jamesi* (n = 17)	1	15	1	0
* An. kawari* (n = 16)	0	16	0	0
* An. kochi* (n = 4)	0	4	0	0
* An. maculatus* (n = 9)	4	5	0	0
* An. minimus* (n = 67)	0	5	0	0
* An. peditaeniatus* (n = 5)	0	5	0	0
* An. philippinnensis* (n = 4)	0	4	0	0
* An. sinensis* (n = 8)	1	7	0	0
* An. splendidus* (n = 21)	1	18	1	1
* An. tessellatus* (n = 1)	0	1	0	0
* An. vagus* (n = 5)	1	4	0	0

### **Prevalence of*Plasmodium*spp**

Dissection of the salivary glands for the detection of *Plasmodium* sporozoites was performed for 1,680 specimens (93% total *Anopheles* specimens captured). No specimen was found positive for *Plasmodium* sporozoites by microscopy. From these, a subset of specimens (n = 400) were further examined for *Plasmodium* infection by PCR. Priority for inclusion in the subsample was given to specimens collected by HDNT, and included a total of seven *Anopheles* spp. (Additional file 1: Table 4). Two *P. vivax* positive specimens were identified by PCR from the Ia Mlah commune of Krong Pa district. Both positive specimens were identified in *An. dirus* mosquitoes captured by HDNT in the forest sites between 20:00–21:00 and 21:00–22:00. Origin of blood detected in the midgut was confirmed as porcine by PCR.

## Discussion

Understanding the vector bionomics and *Plasmodium* spp. transmission role of *Anopheles* spp. is essential to inform effective vector prevention measures for populations at high risk of vector exposure. Using three different mosquito collection techniques, *Plasmodium* vectors in forest and farm sites of two districts of Gia Lai province in the central highlands of Vietnam, where there is ongoing malaria transmission, were investigated [5]. HDNT, ABT and CDC light trap captured *An. aconitus* in the greatest numbers compared with other species, and determined that the peak biting time occurred between the 20:00–21:00. The number *An. dirus* and *An. minimus*, commonly regarded as the primary malaria vectors in the region, was comparatively lower, and only two *Plasmodium* infections could be identified in a sub-analysis of two *An. dirus* specimens, demonstrating a very low prevalence of infected vectors in the region at the time of the survey. The findings of this survey provide valuable insight to inform effective implementation of vector control interventions to further reduce vector exposure and malaria transmission in this region.

Collections made by HDNT demonstrated that the majority of anthropophilic activity in both forest and farm sites occurs after 20:00 when individuals are more likely to be indoors and protected by ITNs. In Duc Co district, no mosquitoes were collected between 4:00 and 17:00, and in Krong Pa there were a small number of mosquitoes collected in the early morning (24:00–6:00), but none made in daytime hours (7:00–18:00). Biting in the early evening hours was observed in both sites, particularly for vectors of concern *An. aconitus* and *An. maculatus* in Duc Co district, and *An. dirus* in Krong Pa district. These findings are consistent with previous surveys conducted within Central Vietnam [5, 8], and demonstrate that in some areas where transmission of *Plasmodium* spp. is ongoing, additional protective measures may be required to prevent early evening biting and to protect high risk populations such as forest goers. Recent evidence has demonstrated that individual level interventions such as topical insect repellents and targeted chemoprophylaxis can reduce the risk of *P. falciparum* infection in high-risk populations [14, 15], and distribution of long-lasting insecticide treated hammocks, which may be a more practical protection method for some forest going populations than traditional ITNs, was shown to substantially reduce total malaria cases in a trial in Central Vietnam [16]. Further, outdoor residual spraying was recently demonstrated to reduce biting rates in regions where primary vectors have a tendency to bite outdoors at times when people are unlikely to be using ITNs [17], and may therefore be a suitable supplement to ITNs to reduce vector exposure in Central Vietnam.

Overall, the number of *Anopheles* spp. captured, and the species composition and diversity were greater in Ia Dom commune compared with Mlah commune, but within each commune, species composition and diversity were consistent across HDNT and CDC light traps regardless of the environment (forest vs. farm). ABT using cattle bait yielded the highest number of collections and breadth of species in both communes, demonstrating a high zoophilic behaviour for many species collected which was also demonstrated through detection of host blood in a subset of captured specimens. Collections made by HDNT were of considerably lower density and breadth of species compared with ABT, perhaps reflecting the greater preference for animal bait by a large proportion of *Anopheles* species captured in this survey. Due to the need for volunteers to enter and spend time in the ABT to make collections, and because no repellent measures were taken, it is possible human collectors may have acted as additional bait for short periods. However, due to the marked difference in the breadth of species collected by human and cattle baited traps we do not expect any human attractant to have significantly contaminated this trapping method. The number of species and density of specimens collected was comparable between HDNT and CDC light trap collection methods, however, hourly collection data was not performed for CDC light traps preventing determination of likely biting times for this method. HDNT provides a safer alternative to more traditional capture techniques such as human landing catch where volunteers collect mosquitoes that land on the exposed limb, thus exposing them to potentially infectious bites. HDNT was useful for collection of anthropophilic species and relevant for determining the frequency of vectors of concern and transmission role in human malaria. While not directly compared in the present study, HDNT has been documented to facilitate catching and estimate human biting rates similar to the gold standard human landing catch for *Anopheles* and *Culex* spp., and to a lesser extent, *Aedes* spp [10, 18, 19].

*Anopheles* spp. considered primary vectors of *Plasmodium* spp., *An. dirus* and *An. minimus*, were found overall at low density compared to secondary vectors such as *An. aconitus*, which was found at the highest density in all sites and by all trapping methods. *Anopheles dirus* has been identified as an important malaria vector in the forest and forest fringe in Southeast Asia and Vietnam, especially during the rainy season. Even at low densities, *An. dirus* is an important vector in the region due to its anthropophilic tendencies and long-life span [20, 21]. Although found in low numbers, *An. dirus* was the only species infected with *Plasmodium* spp. assessed in a subsample of specimens (including seven out of the 19 total species collected in this study). Interestingly, despite *P. falciparum* infections making up the majority of reported malaria cases in both communes preceding this survey, both of the two *Plasmodium* spp. positive *An. dirus* specimens were infected with *P. vivax*, indicating that *P. vivax* transmission still occurs in Il Mlah commune where these specimens were captured despite a greater proportion of malaria cases being identified as *P. falciparum*. Importantly, both *P. vivax* positive *An. dirus* specimens were captured by HDNT, the most relevant method for capturing anthropophilic mosquitoes utilized in this survey. This finding highlights the importance of continuing to monitor the role of *An. dirus* in *Plasmodium* transmission in this region and to identify biting behaviour that can be prevented with currently available interventions.

Bionomics of primary malaria vectors varies seasonally between species within the central highlands of Vietnam. *An. dirus* and *An. maculatus* numbers are often higher at the end of the rainy season (October–November), while *An. minimus* also thrives at the end of the dry season (April–May) (NIMPE pers. comm.). Whilst the number of *Plasmodium* infected specimens was small, this finding is limited to a single survey lasting twelve days (total 96 person nights) conducted at the end of the wet season/beginning of the dry season due to accessibility issues and COVID-19 related disruptions. As such, these findings are unlikely to reflect total *Anopheles* numbers and their transmission potential in other seasons and provinces. Additional surveys will be required to determine seasonal trends in vector density and their relative importance for malaria transmission. Additionally, due to cost and throughput constraints, it was not possible to perform analyses of *Plasmodium* infection by PCR or determination of host blood meal origin in all captured specimens and instead sub-samples were selected. This likely limited the ability to determine biting preferences and transmission potential for a number of species that were not represented by this sub-sample, and future investigations would benefit from wider and more diverse inclusion of captured species in these types of analyses to ensure malaria vectors of concern are adequately identified and their host seeking preferences well characterized.

## Conclusion

This study identified a small number of vectors of concern and very low numbers of *Plasmodium* infected *Anopheles* mosquitoes at the end of the rainy season in forested and farm sites of the Central Highlands of Vietnam. Capture of *Anopheles* spp. of vectorial importance by HDNT in early evening hours demonstrates that additional vector control and personal protection measures may benefit populations frequenting forest and farm sites to prevent vector exposure when they cannot be protected by ITNs. Additional investigation of vector bionomics and *Plasmodium* transmission role of *Anopheles* spp. is required throughout regions of Vietnam in the malaria pre-elimination phase to inform control interventions to further reduce vector exposure and malaria transmission.

## Supplementary Information

**Additional file 1: Table S1.** qPCR primer and probe sequences for Plasmodium spp. detection. **Table S2.** qPCR assay specifications. **Table S3.** Genus composition of collected mosquitoes (n). **Table S4.** Species and trapping method of specimens analysed by PCR and diffused serum agglutination. **Figure S1.** Location of Gia Lai Province, Vietnam, and location of Ia Dom and Ia Mlah communes within Duc Co and Krong Pa Districts, Gia Lai Province. **Figure S2.** Number of Anopheles mosquitoes collected nightly by HDNT in farm and forest sites in Krong Pa District by species. Each connected line represents a separate collection night. **Figure S3.** Number of Anopheles mosquitoes collected nightly by HDNT in farm and forest sites in Krong Pa District by species. Each connected line represents a separate collection night.

## Data Availability

The datasets used during the current study are available from the corresponding author on reasonable request.

## Abbreviations

ABT: Animal baited trap
CDC: Centers for disease control
HDNT: Human baited double net trap
LT: Light trap
NIMPE: National Institute of Malariology, Parasitology and Entomology
PCR: Polymerase chain reaction
