# Supplementary material for: Anopheles diversity, biting behaviour and transmission potential in forest and farm environments of Gia Lai province, Vietnam
Source: Malar J. 2023 Jul 5;22:204. doi: 10.1186/s12936-023-04631-1 (PMC10320868; doi:10.1186/s12936-023-04631-1)
Supplement: Supplementary file 1 — Additional file 1: Table S1. qPCR primer and probe sequences for Plasmodium spp. detection. Table S2. qPCR assay specifications. Table S3. Genus composition of collected mosquitoes (n). Table S4. Species and trapping method of specimens analysed by PCR and diffused serum agglutination. Figure S1. Location of Gia Lai Province, Vietnam, and location of Ia Dom and Ia Mlah communes within Duc Co and Krong Pa Districts, Gia Lai Province. Figure S2. Number of Anopheles mosquitoes collected nightly by HDNT in farm and forest sites in Krong Pa District by species. Each connected line represents a separate collection night. Figure S3. Number of Anopheles mosquitoes collected nightly by HDNT in farm and forest sites in Krong Pa District by species. Each connected line represents a separate collection night. [file 12936_2023_4631_MOESM1_ESM.docx]

**Additional material**

| **Additional Table 1: qPCR primer and probe sequences for *Plasmodium* spp. detection** | | |
| --- | --- | --- |
| ***Plasmodium* spp.** | **Primer/Probe** | **Sequences** |
|  | PAN- R | TGTTGAGTCAAATTAAGCCGCAA |
|  | PAN- F | TTAGATTGCTTCCTTCAGTRCCTTATG |
|  | PAN- Probe | FAM-TCAATTCTTTTAACTTTCTCGCTTGCGCGA-BHQ1 |
| ***P. falciparum*** |  |  |
|  | Fal-F | CTCTTCAATATGCTTTTATTGCTTTTGAGA |
|  | Fal-R | GCTTATTCATATTTGTTATTCCATGCTGTA |
|  | Fal-Probe | FAM-ACAATGAACTCAATCATGACTACCC-BHQ1 |
| ***P. vivax*** |  |  |
|  | Vix-F | ACGCTTCTAGCTTAATCCACATAACT |
|  | Viv-R | TAATTTACTCAAAGTAACAAGGACTTCCAA |
|  | Vix-Probe | HEX-TCGTATCGACTTTGTGCGCATTTTGT-BHQ1 |
| ***P. malariae*** |  |  |
|  | Mal-F | GTAATGCTTTGTATATTTATAACAAAGTTG |
|  | Mal-R | CTCAAAGTAACAAAATTCCCATGCATAA |
|  | Mal-Probe | NED - ATAATAAGAACGCAT - MGB NFQ |
| ***P. ovale*** |  |  |
|  | Oval-F | ATACMACGTATCTGYTCTTTGC |
|  | Oval-R | ACTCAAAGTAACAAAATCTCCWGTAA |
|  | Oval-Probe | VIC- TCCAAAATGTGTTCTTATTA - MGB NFQ |
| ***P. knowlesi*** |  |  |
|  | Know-F | GCATCATAATCCAGTTTTATG |
|  | Know-R | TACCTTGTACCTAATAATACTTGG |
|  | Know-Probe | FAM-CAGGGAATAGAGGGTTG-MGB NFQ |

| **Additional Table 2: qPCR assay specifications** | |
| --- | --- |
| **Reagents** | **Volume / reaction (µl)** |
| 2X quantinova probe mix | 6.250 |
| PAN/Fal/Viv/Mal/Oval/Know_F (conc work solution 25µM) | 0.175 |
| PAN/Fal/Viv/Mal/Oval/Know _R (conc work solution 25µM) | 0.175 |
| PAN/Fal/Viv/Mal/Oval/Know Probe (conc work solution 10µM) | 0.375 |
| H_2_O | 0.525 |
| DNA sample | 5 |
| Total | 7.5 |
| **Cycling parameters** | |
| Step 1: activation | 95°C, 3min |
| Step 2: amplification (45 cycles) | 95°C, 5sec |
|  | 58°C, 30sec |
| Step 3: Cooling | Hold at 15°C |

| **Additional Table 3: Genus composition of collected mosquitoes (n)** | | |  |
| --- | --- | --- | --- |
| **Genus** | **Ia Dom commune, Duc Co district (N=1,007)** | **Ia Mlah commune, Krong Pa district (N=1,920)** | |
| *Aedes* spp. (N=49) | 2 | 47 | |
| *Anopheles* spp. (N=1,815) | 870 | 945 | |
| *Culex* spp. (n=1,060) | 132 | 928 | |
| *Mansonia* spp. (N=3) | 3 | 0 | |

| **Additional Table 4: Species and trapping method of specimens analysed by PCR and diffused serum agglutination** | | | | | | | | | | |
| --- | --- | --- | --- | --- | --- | --- | --- | --- | --- | --- |
| ***Anopheles***  **spp.** | **Ia Dom commune, Duc Co district - N mosquitoes** | | | | | **Ia Mlah commune, Krong Pa district - N mosquitoes** | | | | |
|  | **Forest** | | **Farm** | | | **Forest** | | **Farm** | | |
|  | **HDNT** | **LT** | **HDNT** | **LT** | **ABT** | **HDNT** | **LT** | **HDNT** | **LT** | **ABT** |
| ***Plasmodium* spp. infection and blood meal origin determined by PCR (n=400)** | | | | | | | | | | |
| *An. dirus* |  | 1 |  |  |  | 20 | 5 | 3 | 7 |  |
| *An. minimus* | 1 |  | 13 | 13 | 37 |  |  | 3 |  |  |
| *An. aconitus* | 13 | 12 | 14 | 24 | 4 | 63 | 20 | 50 | 14 | 3 |
| *An. jeyporiensis* |  |  |  |  |  |  |  |  |  | 2 |
| *An. maculatus* | 17 | 2 | 8 | 6 | 14 | 7 | 1 | 11 | 1 | 1 |
| *An. sinensis* |  |  | 1 |  |  |  |  |  |  |  |
| *An. splendidus* |  |  |  |  |  | 2 |  |  | 7 |  |
| **Blood meal origin determined by diffused serum agglutination (n=200)** | | | | | | | | | | |
| *An. aconitus* | 3 | 1 | 2 | 3 | 25 | 4 | 5 | 13 | 8 | 36 |
| *An. barbirostris* |  |  |  |  | 1 |  |  |  |  |  |
| *An. dirus* |  |  |  |  |  |  | 3 |  | 1 |  |
| *An. jamesi* |  |  |  |  | 11 |  |  |  |  | 6 |
| *An. kawari* |  |  |  |  | 16 |  |  |  |  |  |
| *An. kochi* |  |  |  |  | 1 |  |  |  |  | 3 |
| *An. maculatus* | 2 | 1 | 1 | 0 | 3 |  |  |  |  | 2 |
| *An. minimus* |  |  | 2 | 1 | 2 |  |  |  |  |  |
| *An. peditaeniatus* | 1 |  |  |  | 4 |  |  |  |  |  |
| *An. philippinnensis* |  |  | 1 |  | 2 |  |  |  | 1 |  |
| *An. sinensis* | 1 |  |  |  | 5 |  |  |  |  | 2 |
| *An. splendidus* |  |  |  |  | 14 |  |  |  | 2 | 5 |
| *An. tessellatus* |  |  |  |  |  |  |  |  |  | 1 |
| *An. vagus* |  |  |  |  |  |  |  |  |  | 5 |
| HDNT: human baited double net trap, ABT: animal baited trap, LT: light trap | | | | | | | | | | |

**
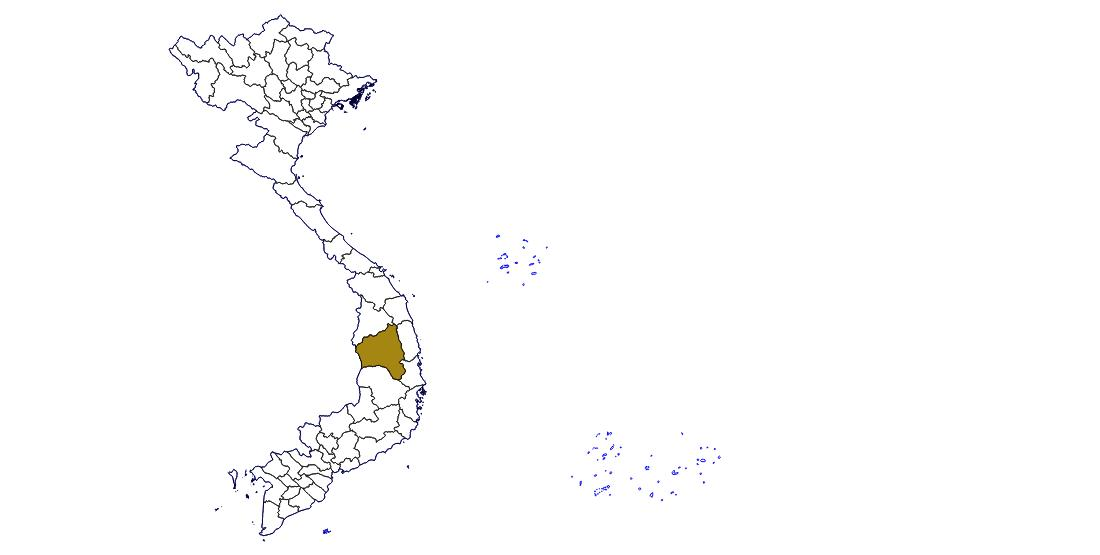

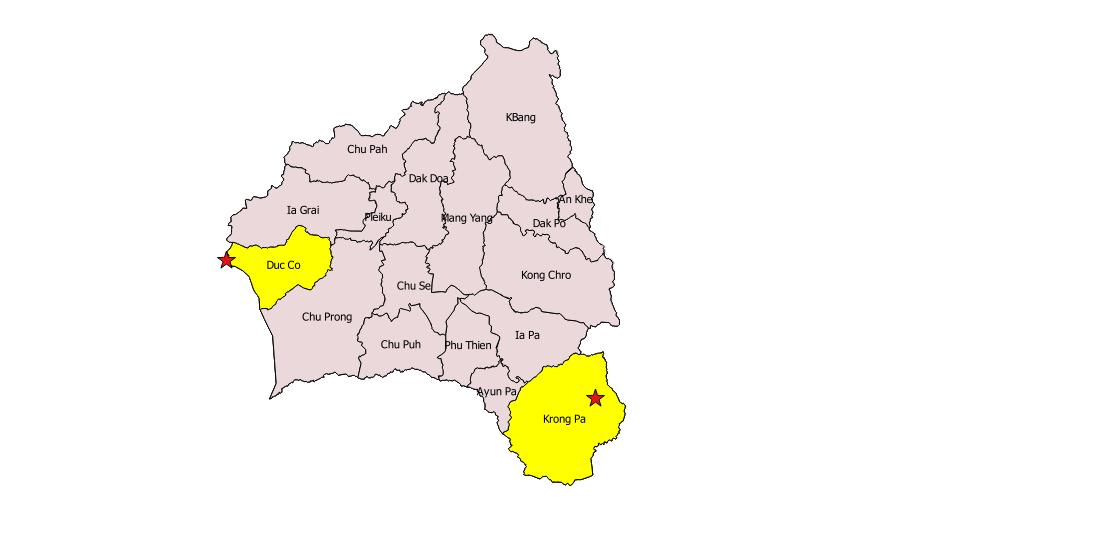
**

**Additional Figure 1**: Location of Gia Lai Province, Vietnam, and location of Ia Dom and Ia Mlah communes within Duc Co and Krong Pa Districts, Gia Lai Province.

**Additional Figure 2:** Number of Anopheles mosquitoes collected nightly by HDNT in farm and forest sites in Krong Pa District by species. Each connected line represents a separate collection night.

**Additional Figure 3:** Number of Anopheles mosquitoes collected nightly by HDNT in farm and forest sites in Krong Pa District by species. Each connected line represents a separate collection night.
